# Diosgenin-3,6-dione: second polymorph in space group *P*2_1_2_1_2_1_

**DOI:** 10.1107/S2414314620012006

**Published:** 2020-09-08

**Authors:** Gabriel Guerrero-Luna, Jaquelin Reyes Melchor, Sylvain Bernès, María-Guadalupe Hernández-Linares

**Affiliations:** a Facultad de Ciencias Químicas, Benemérita Universidad Autónoma de Puebla, 72570 Puebla, Pue., Mexico; b Licenciatura en Biotecnología, Benemérita Universidad Autónoma de Puebla, 72570 Puebla, Pue., Mexico; c Instituto de Física, Benemérita Universidad Autónoma de Puebla, 72570 Puebla, Pue., Mexico; d Centro de Química, Instituto de Ciencias, Benemérita Universidad Autónoma de Puebla, 72570 Puebla, Pue., Mexico; e Laboratorio de Investigación, Herbario y Jardín Botánico Universitario, Benemérita Universidad Autónoma de Puebla, 72570 Puebla, Pue., Mexico; University of Zürich, Switzerland

**Keywords:** crystal structure, steroids, diosgenin, polymorphism

## Abstract

The title steroid, [(25*R*)-spirost-4-en-3,6-dione, C_27_H_38_O_4_], is obtained by oxidation of diosgenin, using the Jones reagent (CrO_3_/H_2_SO_4_). The crystal structure was previously reported in space group *P*2_1_2_1_2_1_, but nonetheless with the wrong absolute configuration and omitting positions for H atoms [Rajnikant *et al.* (2000 ▸). *Mol. Cryst. Liq. Cryst. Sci. Technol. Sect. C*, **12**, 101–110]. The diffraction data set reported herein is for a second polymorph in the same space group, as evidenced by simulated powder patterns. Both forms are characterized by a similar ortho­rhom­bic unit cell, and a similar arrangement of the mol­ecules in the crystal structure. However, the conformation of the *A*/*B* rings in the steroid nucleus is slightly modified, leading to the observed polymorphism.

## Abstract 2

A polymorphic modification of the title steroidal compound, derived from diosgenin, results from a conformational alteration for the *A*/*B* ring system.

## Graphical abstract

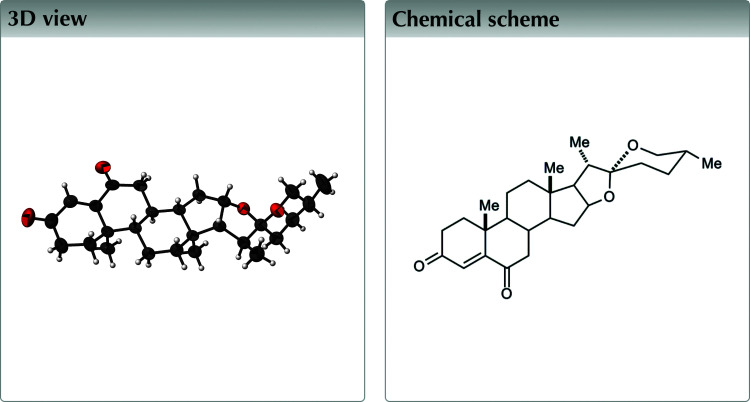

## Structure description

Diosgenin [(3*β*,25*R*)-spirost-5-en-3-ol, C_27_H_42_O_3_] is a natural product that has played a pivotal role in the early stages of the industry of steroidal compounds, including the large-scale synthesis of cortisone, and the manufacturing of the first combined oral contraceptive pills, at Syntex S.A., in Mexico (Djerassi, 1992 ▸). Diosgenin treated with the Jones reagent gives the expected oxidation product, with carbonyl groups at C3 and C6. This compound was characterized by X-ray crystallography, and its structure reported twenty years ago (Rajnikant *et al.*, 2000 ▸). The reported refinement is rather technically unsound, since all H atoms were omitted in the model and the wrong absolute configuration was assigned to the mol­ecule (see refcode QUPKUH in the CSD; Groom *et al.*, 2016 ▸). Since the Jones oxidation does not affect the *E*/*F* rings of diosgenin, chiral centre C25 is expected to retains its original *R* configuration, while the structure currently deposited in the CSD has a 25*S* configuration. A suitable model can be restored after inversion of the structure and addition of H atoms in calculated positions.

**Figure 1:**
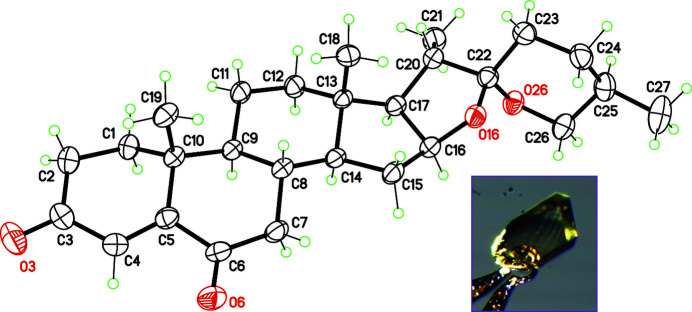
Mol­ecular structure of the title steroid, with displacement ellipsoids for non-H atoms at the 30% probability level. The inset is the crystal used for data collection. The largest dimension is 0.5 mm.

**Figure 2:**
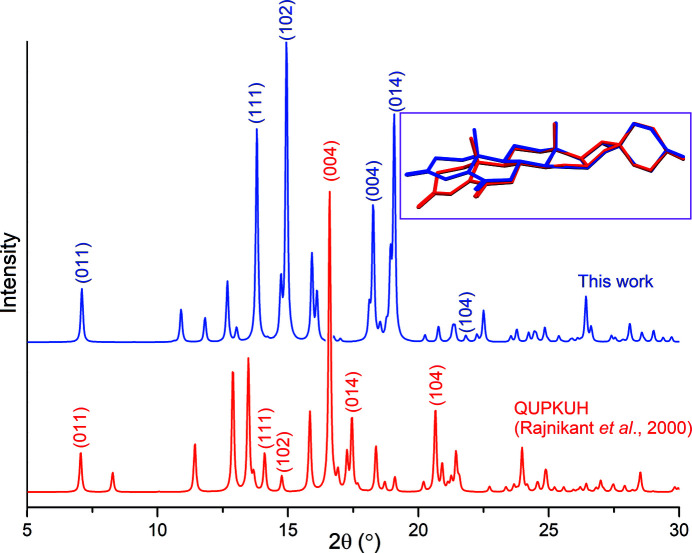
Simulated powder patterns (Macrae *et al.*, 2020 ▸) for the two *P*2_1_2_1_2_1_ polymorphs of the title compound, with λ = 1.54 Å. Some reflections are indexed, in order to illustrate reflection shifts and intensity variations between both polymorphs. The inset shows an overlay between mol­ecular structures, obtained by fitting C and O atoms in rings *C*/*D*/*E*/*F*. In the case of QUPKUH (Rajnikant *et al.*, 2000 ▸), coordinates deposited in the CSD were inverted, and H atoms were placed in idealized positions.

While working with this mol­ecule, we obtained high-quality, well-shaped prismatic crystals (Fig. 1 ▸), and collected diffraction data with the purpose of improving the previously reported structure. However, it soon became clear that a new form had been crystallized instead; although the crystal symmetry was unchanged, differences in cell parameters as large as 2 Å were observed. After structure refinement (Table 1 ▸), a simulated powder diffraction pattern was compared with that obtained with the model of Rajnikant *et al.* (2000 ▸). Patterns are clearly different, as expected for two polymorphic forms (Fig. 2 ▸). The polymorphism seems to be a consequence of a slight modification of the conformation for rings *A* and *B* (Fig. 2 ▸, inset). In the structure reported herein, ring *A* displays a distorted envelope conformation, with a puckering amplitude *q* = 0.458 (3) Å, and ring *B* is a distorted half-chair, with *q* = 0.476 (3) Å. For the previously characterized polymorph, ring *A* is nearest to an half-chair, and *B* to a chair conformation. The conformational flexibility of the *A* ring of steroids bearing a conjugated 4-ene-3,6-dione fragment had already been pointed out (Anthony *et al.*, 1998 ▸), and related to the modulation of the steroid-receptor inter­actions, which control hormonal responses for these mol­ecules (Duax *et al.*, 1994 ▸).

**Figure 3:**
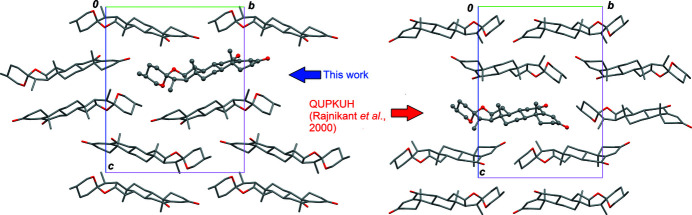
Comparison of the crystal structure for the new polymorph (left) with that previously reported (right). One mol­ecule is chosen arbitrarily as the asymmetric unit and displayed in ball-and-stick style, in order to have roughly the same orientation with respect to cell axis in both forms. Projections are viewed down crystallographic *a* axis.

Since the space group is unchanged, there is a degree of similarity between the crystal structures for the polymorphic forms: the mol­ecules, placed in general positions, lie approximately parallel to [010]. However, if a common orientation is chosen for the asymmetric units in both forms, the position of the mol­ecule in the unit cell is shifted. With the selection made in Fig. 3 ▸, the centroid of the mol­ecule constituting the asymmetric unit for the form reported herein is found at (0.313, 0.661, 0.380), while for the previously reported form, the centroid lies at (0.951, 1/5, 0.628). The action of the screw axis of space group *P*2_1_2_1_2_1_ then generates different crystal structures. This kind of polymorphism, resulting from the rearrangement of the asymmetric unit within a common space group, is certainly favoured by the lack of supra­molecular inter­actions. The title mol­ecule does not include donor groups for hydrogen bonds, and only very weak C—H⋯O contacts are present in the crystals. A closely related type of polymorphism in space group *P*2_1_ was reported for diosgenone [(25*R*)-spirost-4-en-3-one, C_27_H_40_O_3_; Hernández Linares *et al.*, 2012 ▸], which crystallizes with *Z*′ = 2. In that case, one of the independent mol­ecules in the asymmetric unit changes its orientation, and unit-cell parameters vary considerably between polymorphs, even though crystal symmetry is retained.

## Synthesis and crystallization

To a solution of diosgenin (2.0 g, 4.8 mmol) in 20 mL of CH_2_Cl_2_ and 40 mL of acetone was slowly added a solution of Jones reagent (10 mL: 1.8 g, 18.4 mmol of CrO_3_ in H_2_O/H_2_SO_4_ 8:2) over 10 min in an ice bath. The reaction was kept under stirring at room temperature and monitored by TLC until a change in colour (orange to green) was observed. Subsequently, 2-propanol was added to quench unreacted Jones reagent, and the reaction mixture was poured into a separating funnel and extracted with ethyl acetate. The solution was washed with distilled H_2_O, neutralized with NaHCO_3_, separated, dried over Na_2_SO_4_ and evaporated to dryness under reduced pressure. The purification was carried out on a chromatographic column with silica gel (hexa­ne:EtOAc, 9:1), affording 1.63 g (80% yield) of the title compound, while remaining solid was identified as starting material (20%). Single crystals for the compound of inter­est were obtained by slow evaporation of the corresponding chromatographic fraction.

## Refinement

Crystal data, data collection and structure refinement details are summarized in Table 1 ▸.

## Supplementary Material

Crystal structure: contains datablock(s) I, global. DOI: 10.1107/S2414314620012006/zq4041sup1.cif

Structure factors: contains datablock(s) I. DOI: 10.1107/S2414314620012006/zq4041Isup2.hkl

CCDC reference: 2026501

Additional supporting information:  crystallographic information; 3D view; checkCIF report

## Figures and Tables

**Table 1:** Experimental details

Crystal data	
Chemical formula	C_27_H_38_O_4_
*M*_r_	426.57
Crystal system, space group	Orthorhombic, *P*2_1_2_1_2_1_
Temperature (K)	295
*a*, *b*, *c* (Å)	7.4768 (3), 16.2190 (9), 19.4101 (10)
*V* (Å^3^)	2353.8 (2)
*Z*	4
Radiation type	Ag *K*α, λ = 0.56083 Å
μ (mm^−1^)	0.05
Crystal size (mm)	0.50 × 0.40 × 0.15

Data collection	
Diffractometer	Stoe Stadivari
Absorption correction	Multi-scan (*X-AREA*; Stoe & Cie, 2018 ▸)
*T*_min_, *T*_max_	0.471, 1.000
No. of measured, independent and observed [*I* > 2σ(*I*)] reflections	47703, 5150, 3345
*R*_int_	0.073
(sin θ/λ)_max_ (Å^−1^)	0.639

Refinement	
*R*[*F* ^2^ > 2σ(*F* ^2^)], *wR*(*F* ^2^), *S*	0.040, 0.091, 0.88
No. of reflections	5150
No. of parameters	285
H-atom treatment	H-atom parameters constrained
Δρ_max_, Δρ_min_ (e Å^−3^)	0.23, −0.21

Computer programs: *X-AREA* (Stoe & Cie, 2018 ▸), *SHELXT2018/2* (Sheldrick, 2015*a* ▸), *SHELXL2018/3* (Sheldrick, 2015*b* ▸), *XP* in *SHELXTL-Plus* (Sheldrick, 2008 ▸), *Mercury* (Macrae *et al.*, 2020 ▸) and *publCIF* (Westrip, 2010 ▸).

## full crystallographic data

### Crystal data

**Table:**

C_27_H_38_O_4_	*D*_x_ = 1.204 Mg m^−^^3^
*M**_r_* = 426.57	Melting point: 453 K
Orthorhombic, *P*2_1_2_1_2_1_	Ag *K*α radiation, λ = 0.56083 Å
*a* = 7.4768 (3) Å	Cell parameters from 25730 reflections
*b* = 16.2190 (9) Å	θ = 2.3–25.0°
*c* = 19.4101 (10) Å	µ = 0.05 mm^−^^1^
*V* = 2353.8 (2) Å^3^	*T* = 295 K
*Z* = 4	Prism, yellow
*F*(000) = 928	0.50 × 0.40 × 0.15 mm

### Data collection

**Table:**

Stoe Stadivari diffractometer	5150 independent reflections
Radiation source: Sealed X-ray tube, Axo Astix-f Microfocus source	3345 reflections with *I* > 2σ(*I*)
Graded multilayer mirror monochromator	*R*_int_ = 0.073
Detector resolution: 5.81 pixels mm^-1^	θ_max_ = 21.0°, θ_min_ = 2.3°
ω scans	*h* = −9→9
Absorption correction: multi-scan (X-AREA; Stoe & Cie, 2018)	*k* = −20→20
*T*_min_ = 0.471, *T*_max_ = 1.000	*l* = −24→24
47703 measured reflections	

### Refinement

**Table:**

Refinement on *F*^2^	Secondary atom site location: difference Fourier map
Least-squares matrix: full	Hydrogen site location: inferred from neighbouring sites
*R*[*F*^2^ > 2σ(*F*^2^)] = 0.040	H-atom parameters constrained
*wR*(*F*^2^) = 0.091	*w* = 1/[σ^2^(*F*_o_^2^) + (0.0488*P*)^2^] where *P* = (*F*_o_^2^ + 2*F*_c_^2^)/3
*S* = 0.88	(Δ/σ)_max_ < 0.001
5150 reflections	Δρ_max_ = 0.23 e Å^−^^3^
285 parameters	Δρ_min_ = −0.21 e Å^−^^3^
0 restraints	Extinction correction: SHELXL-2018/3 (Sheldrick 2015b), Fc^*^=kFc[1+0.001xFc^2^λ^3^/sin(2θ)]^-1/4^
0 constraints	Extinction coefficient: 0.0162 (19)
Primary atom site location: dual	

### Special details

**Table:**

Refinement. All H atoms were placed in calculated positions and refined as riding to their carrier C-atoms (Sheldrick, 2015*b*).

### Fractional atomic coordinates and isotropic or equivalent isotropic displacement parameters (Å^2^)

**Table:**

	*x*	*y*	*z*	*U*_iso_*/*U*_eq_	
C1	0.0439 (3)	0.97069 (16)	0.37910 (14)	0.0522 (7)	
H1A	−0.071025	0.943263	0.382619	0.063*	
H1B	0.090567	0.977185	0.425417	0.063*	
C2	0.0159 (4)	1.05554 (17)	0.34782 (16)	0.0606 (7)	
H2A	−0.060529	1.050286	0.307687	0.073*	
H2B	−0.045802	1.090035	0.381014	0.073*	
C3	0.1843 (4)	1.09697 (19)	0.32713 (19)	0.0755 (9)	
O3	0.1874 (4)	1.16980 (15)	0.3114 (2)	0.1337 (13)	
C4	0.3462 (4)	1.04615 (18)	0.32287 (16)	0.0643 (8)	
H4	0.455697	1.072524	0.317233	0.077*	
C5	0.3444 (3)	0.96390 (17)	0.32668 (13)	0.0479 (6)	
C6	0.5191 (3)	0.91992 (17)	0.31852 (13)	0.0504 (7)	
O6	0.6482 (2)	0.95624 (13)	0.29488 (12)	0.0731 (6)	
C7	0.5344 (3)	0.83377 (17)	0.34334 (15)	0.0548 (7)	
H7A	0.585728	0.834932	0.389224	0.066*	
H7B	0.618289	0.805013	0.313743	0.066*	
C8	0.3615 (3)	0.78329 (14)	0.34624 (13)	0.0423 (6)	
H8	0.326505	0.768461	0.299195	0.051*	
C9	0.2114 (3)	0.83575 (14)	0.37883 (12)	0.0406 (6)	
H9	0.256600	0.853585	0.423812	0.049*	
C10	0.1720 (3)	0.91550 (15)	0.33790 (12)	0.0415 (6)	
C11	0.0418 (3)	0.78446 (15)	0.39364 (15)	0.0518 (7)	
H11A	−0.041358	0.817845	0.419956	0.062*	
H11B	−0.015180	0.770631	0.350262	0.062*	
C12	0.0794 (3)	0.70518 (16)	0.43324 (14)	0.0513 (7)	
H12A	0.122140	0.718791	0.479036	0.062*	
H12B	−0.030678	0.674098	0.438101	0.062*	
C13	0.2181 (3)	0.65227 (14)	0.39675 (12)	0.0394 (6)	
C14	0.3879 (3)	0.70496 (15)	0.38761 (13)	0.0420 (6)	
H14	0.421477	0.722961	0.434037	0.050*	
C15	0.5293 (3)	0.64196 (16)	0.36631 (15)	0.0549 (7)	
H15A	0.517289	0.627142	0.318116	0.066*	
H15B	0.649095	0.662747	0.374435	0.066*	
C16	0.4875 (3)	0.56896 (16)	0.41304 (14)	0.0499 (6)	
H16	0.572569	0.566620	0.451408	0.060*	
O16	0.4847 (2)	0.49248 (10)	0.37698 (9)	0.0516 (5)	
C17	0.2937 (3)	0.58071 (16)	0.44046 (13)	0.0450 (6)	
H17	0.298534	0.597779	0.488857	0.054*	
C18	0.1451 (3)	0.62202 (17)	0.32730 (13)	0.0537 (7)	
H18A	0.033182	0.594318	0.334407	0.081*	
H18B	0.229110	0.584524	0.306835	0.081*	
H18C	0.127487	0.668280	0.297204	0.081*	
C19	0.0918 (3)	0.89664 (18)	0.26624 (13)	0.0535 (7)	
H19A	−0.024407	0.872328	0.271690	0.080*	
H19B	0.168538	0.858988	0.242108	0.080*	
H19C	0.081458	0.946891	0.240411	0.080*	
C20	0.2104 (3)	0.49410 (16)	0.43591 (14)	0.0498 (7)	
H20	0.123711	0.494455	0.398032	0.060*	
C21	0.1124 (4)	0.46656 (19)	0.50102 (16)	0.0696 (9)	
H21A	0.192388	0.469427	0.539642	0.104*	
H21B	0.071525	0.410860	0.495363	0.104*	
H21C	0.011807	0.502070	0.508975	0.104*	
C22	0.3684 (3)	0.43940 (16)	0.41412 (14)	0.0477 (6)	
C23	0.3208 (3)	0.36802 (17)	0.36779 (15)	0.0570 (7)	
H23A	0.270637	0.388837	0.325116	0.068*	
H23B	0.230978	0.334086	0.390046	0.068*	
C24	0.4848 (4)	0.31596 (18)	0.35199 (15)	0.0627 (8)	
H24A	0.449256	0.267618	0.325940	0.075*	
H24B	0.567501	0.347584	0.324002	0.075*	
C25	0.5769 (4)	0.28929 (18)	0.41780 (16)	0.0649 (8)	
H25	0.496066	0.252466	0.442952	0.078*	
C26	0.6121 (4)	0.36431 (18)	0.46214 (15)	0.0629 (8)	
H26A	0.663372	0.346687	0.505617	0.076*	
H26B	0.698909	0.399318	0.439267	0.076*	
O26	0.4536 (2)	0.41104 (11)	0.47543 (9)	0.0548 (5)	
C27	0.7500 (5)	0.2427 (2)	0.4031 (2)	0.0991 (13)	
H27A	0.725690	0.197330	0.372742	0.149*	
H27B	0.798657	0.222111	0.445573	0.149*	
H27C	0.834658	0.279181	0.381880	0.149*	

### Atomic displacement parameters (Å^2^)

**Table:**

	*U*^11^	*U*^22^	*U*^33^	*U*^12^	*U*^13^	*U*^23^
C1	0.0547 (13)	0.0515 (16)	0.0503 (16)	0.0115 (12)	0.0024 (13)	−0.0007 (13)
C2	0.0636 (16)	0.0550 (17)	0.0631 (18)	0.0144 (14)	0.0016 (14)	0.0002 (15)
C3	0.074 (2)	0.0483 (19)	0.104 (3)	−0.0016 (15)	−0.0102 (19)	−0.0028 (18)
O3	0.0990 (19)	0.0509 (15)	0.251 (4)	−0.0007 (14)	0.004 (2)	0.020 (2)
C4	0.0515 (14)	0.0590 (19)	0.082 (2)	−0.0071 (13)	−0.0075 (14)	0.0043 (17)
C5	0.0440 (12)	0.0564 (16)	0.0432 (16)	−0.0002 (11)	−0.0065 (11)	0.0027 (13)
C6	0.0366 (12)	0.0634 (18)	0.0514 (16)	−0.0059 (12)	−0.0057 (11)	0.0028 (13)
O6	0.0471 (10)	0.0768 (14)	0.0954 (16)	−0.0070 (10)	0.0063 (10)	0.0133 (13)
C7	0.0341 (11)	0.0633 (18)	0.0669 (18)	0.0033 (11)	0.0019 (12)	0.0072 (15)
C8	0.0340 (11)	0.0520 (15)	0.0409 (14)	0.0035 (10)	−0.0014 (10)	−0.0007 (12)
C9	0.0375 (11)	0.0466 (15)	0.0378 (14)	0.0042 (10)	−0.0018 (10)	−0.0049 (12)
C10	0.0379 (11)	0.0494 (15)	0.0373 (14)	0.0045 (10)	−0.0013 (10)	−0.0007 (12)
C11	0.0379 (11)	0.0494 (15)	0.0682 (18)	0.0069 (11)	0.0106 (12)	0.0001 (14)
C12	0.0440 (13)	0.0485 (16)	0.0615 (18)	0.0040 (11)	0.0125 (12)	−0.0017 (14)
C13	0.0333 (10)	0.0446 (14)	0.0402 (14)	0.0035 (10)	0.0002 (10)	−0.0049 (12)
C14	0.0353 (11)	0.0483 (15)	0.0424 (15)	0.0027 (10)	−0.0042 (10)	−0.0033 (12)
C15	0.0358 (11)	0.0571 (17)	0.0718 (19)	0.0073 (11)	0.0039 (12)	0.0051 (15)
C16	0.0401 (12)	0.0511 (16)	0.0584 (16)	0.0047 (12)	−0.0068 (12)	−0.0021 (14)
O16	0.0461 (9)	0.0493 (11)	0.0593 (11)	0.0051 (8)	0.0042 (8)	−0.0001 (9)
C17	0.0418 (11)	0.0505 (15)	0.0426 (14)	0.0018 (11)	−0.0016 (11)	−0.0027 (12)
C18	0.0539 (14)	0.0549 (16)	0.0524 (17)	−0.0021 (13)	−0.0117 (12)	−0.0033 (13)
C19	0.0458 (13)	0.0684 (18)	0.0462 (16)	0.0077 (12)	−0.0069 (12)	−0.0036 (14)
C20	0.0444 (13)	0.0497 (16)	0.0553 (17)	0.0040 (12)	−0.0008 (12)	−0.0018 (14)
C21	0.0601 (16)	0.0624 (19)	0.086 (2)	0.0003 (14)	0.0207 (16)	0.0036 (17)
C22	0.0464 (12)	0.0491 (16)	0.0478 (15)	0.0022 (12)	−0.0022 (12)	0.0003 (13)
C23	0.0549 (14)	0.0574 (17)	0.0586 (18)	−0.0007 (13)	−0.0019 (13)	−0.0072 (15)
C24	0.0678 (18)	0.0545 (17)	0.0657 (19)	0.0029 (14)	0.0051 (15)	−0.0107 (15)
C25	0.0748 (19)	0.0535 (18)	0.066 (2)	0.0132 (14)	0.0097 (16)	0.0111 (16)
C26	0.0606 (16)	0.067 (2)	0.061 (2)	0.0149 (15)	−0.0047 (14)	0.0072 (16)
O26	0.0579 (10)	0.0583 (11)	0.0483 (11)	0.0113 (9)	−0.0048 (9)	0.0006 (9)
C27	0.111 (3)	0.092 (3)	0.095 (3)	0.052 (2)	0.007 (2)	0.011 (2)

### Geometric parameters (Å, º)

**Table:**

C1—C2	1.519 (4)	C15—H15A	0.9700
C1—C10	1.536 (3)	C15—H15B	0.9700
C1—H1A	0.9700	C16—O16	1.424 (3)
C1—H1B	0.9700	C16—C17	1.556 (3)
C2—C3	1.483 (4)	C16—H16	0.9800
C2—H2A	0.9700	O16—C22	1.420 (3)
C2—H2B	0.9700	C17—C20	1.539 (4)
C3—O3	1.220 (4)	C17—H17	0.9800
C3—C4	1.467 (4)	C18—H18A	0.9600
C4—C5	1.336 (4)	C18—H18B	0.9600
C4—H4	0.9300	C18—H18C	0.9600
C5—C6	1.497 (3)	C19—H19A	0.9600
C5—C10	1.525 (3)	C19—H19B	0.9600
C6—O6	1.220 (3)	C19—H19C	0.9600
C6—C7	1.482 (4)	C20—C21	1.528 (4)
C7—C8	1.531 (3)	C20—C22	1.537 (3)
C7—H7A	0.9700	C20—H20	0.9800
C7—H7B	0.9700	C21—H21A	0.9600
C8—C14	1.516 (3)	C21—H21B	0.9600
C8—C9	1.544 (3)	C21—H21C	0.9600
C8—H8	0.9800	C22—O26	1.426 (3)
C9—C11	1.543 (3)	C22—C23	1.508 (3)
C9—C10	1.546 (3)	C23—C24	1.520 (4)
C9—H9	0.9800	C23—H23A	0.9700
C10—C19	1.545 (3)	C23—H23B	0.9700
C11—C12	1.524 (3)	C24—C25	1.514 (4)
C11—H11A	0.9700	C24—H24A	0.9700
C11—H11B	0.9700	C24—H24B	0.9700
C12—C13	1.521 (3)	C25—C26	1.513 (4)
C12—H12A	0.9700	C25—C27	1.526 (4)
C12—H12B	0.9700	C25—H25	0.9800
C13—C18	1.535 (3)	C26—O26	1.430 (3)
C13—C14	1.540 (3)	C26—H26A	0.9700
C13—C17	1.545 (3)	C26—H26B	0.9700
C14—C15	1.527 (3)	C27—H27A	0.9600
C14—H14	0.9800	C27—H27B	0.9600
C15—C16	1.524 (4)	C27—H27C	0.9600
			
C2—C1—C10	113.9 (2)	C14—C15—H15B	111.3
C2—C1—H1A	108.8	H15A—C15—H15B	109.2
C10—C1—H1A	108.8	O16—C16—C15	112.8 (2)
C2—C1—H1B	108.8	O16—C16—C17	105.13 (19)
C10—C1—H1B	108.8	C15—C16—C17	107.42 (19)
H1A—C1—H1B	107.7	O16—C16—H16	110.4
C3—C2—C1	113.7 (2)	C15—C16—H16	110.4
C3—C2—H2A	108.8	C17—C16—H16	110.4
C1—C2—H2A	108.8	C22—O16—C16	106.70 (18)
C3—C2—H2B	108.8	C20—C17—C13	120.4 (2)
C1—C2—H2B	108.8	C20—C17—C16	104.2 (2)
H2A—C2—H2B	107.7	C13—C17—C16	104.2 (2)
O3—C3—C4	120.9 (3)	C20—C17—H17	109.1
O3—C3—C2	121.5 (3)	C13—C17—H17	109.1
C4—C3—C2	117.5 (3)	C16—C17—H17	109.1
C5—C4—C3	123.3 (3)	C13—C18—H18A	109.5
C5—C4—H4	118.3	C13—C18—H18B	109.5
C3—C4—H4	118.3	H18A—C18—H18B	109.5
C4—C5—C6	117.4 (2)	C13—C18—H18C	109.5
C4—C5—C10	122.0 (2)	H18A—C18—H18C	109.5
C6—C5—C10	120.5 (2)	H18B—C18—H18C	109.5
O6—C6—C7	121.1 (2)	C10—C19—H19A	109.5
O6—C6—C5	120.0 (2)	C10—C19—H19B	109.5
C7—C6—C5	118.8 (2)	H19A—C19—H19B	109.5
C6—C7—C8	116.8 (2)	C10—C19—H19C	109.5
C6—C7—H7A	108.1	H19A—C19—H19C	109.5
C8—C7—H7A	108.1	H19B—C19—H19C	109.5
C6—C7—H7B	108.1	C21—C20—C22	115.3 (2)
C8—C7—H7B	108.1	C21—C20—C17	114.4 (2)
H7A—C7—H7B	107.3	C22—C20—C17	103.40 (19)
C14—C8—C7	110.98 (19)	C21—C20—H20	107.8
C14—C8—C9	109.83 (19)	C22—C20—H20	107.8
C7—C8—C9	109.53 (19)	C17—C20—H20	107.8
C14—C8—H8	108.8	C20—C21—H21A	109.5
C7—C8—H8	108.8	C20—C21—H21B	109.5
C9—C8—H8	108.8	H21A—C21—H21B	109.5
C11—C9—C8	112.12 (19)	C20—C21—H21C	109.5
C11—C9—C10	112.97 (17)	H21A—C21—H21C	109.5
C8—C9—C10	112.89 (19)	H21B—C21—H21C	109.5
C11—C9—H9	106.1	O16—C22—O26	110.21 (18)
C8—C9—H9	106.1	O16—C22—C23	107.9 (2)
C10—C9—H9	106.1	O26—C22—C23	110.8 (2)
C5—C10—C1	107.5 (2)	O16—C22—C20	105.09 (19)
C5—C10—C19	107.54 (19)	O26—C22—C20	107.5 (2)
C1—C10—C19	110.02 (19)	C23—C22—C20	115.2 (2)
C5—C10—C9	110.05 (18)	C22—C23—C24	110.9 (2)
C1—C10—C9	109.80 (19)	C22—C23—H23A	109.5
C19—C10—C9	111.8 (2)	C24—C23—H23A	109.5
C12—C11—C9	113.40 (19)	C22—C23—H23B	109.5
C12—C11—H11A	108.9	C24—C23—H23B	109.5
C9—C11—H11A	108.9	H23A—C23—H23B	108.0
C12—C11—H11B	108.9	C25—C24—C23	110.8 (2)
C9—C11—H11B	108.9	C25—C24—H24A	109.5
H11A—C11—H11B	107.7	C23—C24—H24A	109.5
C13—C12—C11	111.5 (2)	C25—C24—H24B	109.5
C13—C12—H12A	109.3	C23—C24—H24B	109.5
C11—C12—H12A	109.3	H24A—C24—H24B	108.1
C13—C12—H12B	109.3	C26—C25—C24	109.2 (2)
C11—C12—H12B	109.3	C26—C25—C27	110.9 (3)
H12A—C12—H12B	108.0	C24—C25—C27	111.7 (3)
C12—C13—C18	110.30 (19)	C26—C25—H25	108.3
C12—C13—C14	107.60 (19)	C24—C25—H25	108.3
C18—C13—C14	111.7 (2)	C27—C25—H25	108.3
C12—C13—C17	114.7 (2)	O26—C26—C25	112.6 (2)
C18—C13—C17	111.9 (2)	O26—C26—H26A	109.1
C14—C13—C17	100.30 (17)	C25—C26—H26A	109.1
C8—C14—C15	120.5 (2)	O26—C26—H26B	109.1
C8—C14—C13	114.77 (18)	C25—C26—H26B	109.1
C15—C14—C13	103.32 (19)	H26A—C26—H26B	107.8
C8—C14—H14	105.7	C22—O26—C26	113.00 (19)
C15—C14—H14	105.7	C25—C27—H27A	109.5
C13—C14—H14	105.7	C25—C27—H27B	109.5
C16—C15—C14	102.52 (19)	H27A—C27—H27B	109.5
C16—C15—H15A	111.3	C25—C27—H27C	109.5
C14—C15—H15A	111.3	H27A—C27—H27C	109.5
C16—C15—H15B	111.3	H27B—C27—H27C	109.5
			
C10—C1—C2—C3	−47.1 (3)	C17—C13—C14—C8	−179.5 (2)
C1—C2—C3—O3	−168.3 (4)	C12—C13—C14—C15	167.6 (2)
C1—C2—C3—C4	14.7 (4)	C18—C13—C14—C15	−71.3 (2)
O3—C3—C4—C5	−166.9 (4)	C17—C13—C14—C15	47.4 (2)
C2—C3—C4—C5	10.1 (5)	C8—C14—C15—C16	−170.6 (2)
C3—C4—C5—C6	177.4 (3)	C13—C14—C15—C16	−40.9 (2)
C3—C4—C5—C10	−1.7 (5)	C14—C15—C16—O16	133.7 (2)
C4—C5—C6—O6	−15.3 (4)	C14—C15—C16—C17	18.3 (3)
C10—C5—C6—O6	163.9 (2)	C15—C16—O16—C22	−153.07 (18)
C4—C5—C6—C7	161.2 (3)	C17—C16—O16—C22	−36.3 (2)
C10—C5—C6—C7	−19.6 (3)	C12—C13—C17—C20	94.0 (3)
O6—C6—C7—C8	−157.6 (3)	C18—C13—C17—C20	−32.6 (3)
C5—C6—C7—C8	25.9 (4)	C14—C13—C17—C20	−151.1 (2)
C6—C7—C8—C14	−166.6 (2)	C12—C13—C17—C16	−149.8 (2)
C6—C7—C8—C9	−45.2 (3)	C18—C13—C17—C16	83.7 (2)
C14—C8—C9—C11	−49.0 (3)	C14—C13—C17—C16	−34.9 (2)
C7—C8—C9—C11	−171.1 (2)	O16—C16—C17—C20	17.4 (3)
C14—C8—C9—C10	−177.97 (19)	C15—C16—C17—C20	137.7 (2)
C7—C8—C9—C10	59.9 (3)	O16—C16—C17—C13	−109.7 (2)
C4—C5—C10—C1	−29.0 (3)	C15—C16—C17—C13	10.7 (3)
C6—C5—C10—C1	151.8 (2)	C13—C17—C20—C21	−111.6 (3)
C4—C5—C10—C19	89.4 (3)	C16—C17—C20—C21	132.2 (2)
C6—C5—C10—C19	−89.7 (3)	C13—C17—C20—C22	122.2 (2)
C4—C5—C10—C9	−148.6 (3)	C16—C17—C20—C22	6.0 (3)
C6—C5—C10—C9	32.2 (3)	C16—O16—C22—O26	−74.8 (2)
C2—C1—C10—C5	52.6 (3)	C16—O16—C22—C23	164.07 (18)
C2—C1—C10—C19	−64.3 (3)	C16—O16—C22—C20	40.7 (2)
C2—C1—C10—C9	172.3 (2)	C21—C20—C22—O16	−153.3 (2)
C11—C9—C10—C5	178.7 (2)	C17—C20—C22—O16	−27.6 (2)
C8—C9—C10—C5	−52.8 (2)	C21—C20—C22—O26	−35.9 (3)
C11—C9—C10—C1	60.5 (3)	C17—C20—C22—O26	89.8 (2)
C8—C9—C10—C1	−171.03 (19)	C21—C20—C22—C23	88.2 (3)
C11—C9—C10—C19	−61.9 (2)	C17—C20—C22—C23	−146.2 (2)
C8—C9—C10—C19	66.6 (2)	O16—C22—C23—C24	65.5 (3)
C8—C9—C11—C12	50.4 (3)	O26—C22—C23—C24	−55.2 (3)
C10—C9—C11—C12	179.3 (2)	C20—C22—C23—C24	−177.5 (2)
C9—C11—C12—C13	−55.1 (3)	C22—C23—C24—C25	53.6 (3)
C11—C12—C13—C18	−65.2 (3)	C23—C24—C25—C26	−52.4 (3)
C11—C12—C13—C14	56.8 (3)	C23—C24—C25—C27	−175.5 (3)
C11—C12—C13—C17	167.5 (2)	C24—C25—C26—O26	54.8 (3)
C7—C8—C14—C15	−58.7 (3)	C27—C25—C26—O26	178.3 (3)
C9—C8—C14—C15	−179.9 (2)	O16—C22—O26—C26	−61.5 (3)
C7—C8—C14—C13	176.8 (2)	C23—C22—O26—C26	57.9 (3)
C9—C8—C14—C13	55.6 (3)	C20—C22—O26—C26	−175.5 (2)
C12—C13—C14—C8	−59.3 (3)	C25—C26—O26—C22	−58.6 (3)
C18—C13—C14—C8	61.8 (3)		

### Hydrogen-bond geometry (Å, º)

**Table:**

*D*—H···*A*	*D*—H	H···*A*	*D*···*A*	*D*—H···*A*
C23—H23*A*···O6^i^	0.97	2.64	3.474 (4)	144

Symmetry code: (i) −*x*+1, *y*−1/2, −*z*+1/2.
